# Analysis of numerical modeling of steady-state modes of methane–hydrogen mixture transportation through a compressor station to reduce CO_2_ emissions

**DOI:** 10.1038/s41598-024-61361-3

**Published:** 2024-05-08

**Authors:** Vadim Fetisov

**Affiliations:** Department of Petroleum Engineering, Empress Catherine II Saint Petersburg Mining University, Saint Petersburg, Russia

**Keywords:** Hydrogen, Compressors, Pipeline, Safety problems, Simulation model, Natural gas grid, Dynamic modeling, Applied mathematics, Energy science and technology, Energy infrastructure

## Abstract

This study presents a mathematical model to evaluate the performance of gas pipelines during hydrogen injection in a gas pipeline-compressor station. The developed model presents the calculation of methane–hydrogen mixture (CH_4_/H_2_) transportation through the compressor station, where the compensation of pressure drops in the mass and energy balance takes place. Simultaneously, in the operation of the centrifugal blower system of gas compressor stations, the emissions of CO_2_ are considered, considering the mixing of gas media and the compression of CH_4_/H_2_. This mathematical model is realized for the pipeline transportation of hydrogen, at which the principle of mixture expansion occurs. The aim is to solve the problem of CO_2_ emissions at compressor stations. The optimization procedure has been formulated using a system of nonlinear algebraic equalities. The research focuses on the adaptation of existing gas transportation systems to CH_4_/H_2_ transportation and the impact of environmental risks on the operation of compressor station equipment. In this case, it is possible to determine the quantitative amount of hydrogen that can be added to natural gas. By solving the problem of finding the inner point of sets using the system of nonlinear algebraic equalities, it is possible to obtain the control parameters for safety control of technological modes of CH_4_/H_2_ mixture transportation. The study findings reveal that the consumption of gas charger and hydrogen was 50.67 and 0.184 kg/s, respectively, and the estimated efficiency resulting from the modified turbine design was 75.1 percent. These results indicate that the equipment operates more efficiently when hydrogen is being transported. The numerical analytical results indicated in this study hold practical significance for design applications. It will assist in identifying and evaluating the restrictions that may develop during the technological, operational, and design stages of decision-making.

## Introduction

The problems of emissions that cause the greenhouse effect and their control through the introduction of energy-efficient technologies in the operation of gas trunkline system facilities such as compressor stations are faced with the need to realize the technological requirements of the energy policy of each country where the gas transmission system for gas supply to consumers is developed. Among the various environmental areas of new energy compounds, hydrogen (H_2_) is one of the most promising energy alternatives among other "unconventional" gases. Most of the studies^1–5^ prove that the transport of methane-hydrogen mixtures (CH_4_/H_2_) in small proportions can be carried out through existing gas pipelines without upgrading the system with H_2_. H_2_ replacement does not significantly affect the hydromechanical constraints of the system; the constraints relate to the tolerances of pipe structural materials, compressor station equipment, and other elements of the natural gas infrastructure through which CO_2_ is emitted into the atmosphere. This work focuses on the adaptation of existing pipeline systems for gas transport through a compressor station, with the possible conversion of certain system facilities to pure hydrogen transmission to prevent CO_2_ emissions to the atmosphere.

The transition to a situation where hydrogen becomes an important energy carrier requires decades, but great efforts are being made worldwide in the production, delivery, storage, and utilization of hydrogen^6^. From this point of view, analyzing the potential of using real gas pipeline systems for hydrogen delivery is a valid argument. Determining the conditions under which hydrogen can be added to natural gas is the first step of this study. The chemical and physical properties of hydrogen and natural gas differ significantly, which affects the safety of gas transport and use as well as the integrity of the network. In a study carried by Öney et al.^7^, they provided a techno-economic assessment of the pipeline transport of hydrogen, natural gas, and their mixtures in pipeline design, which has a direct bearing on the final present value of the transport. Several studies devoted to the analysis of natural gas flow through gas pipelines using hydrogen mixtures^8–10^ have shown the fact that there are gaps in the study of the hydrogen's effect on the compressor station's system, which pumps natural gas through the pipeline. Therefore Obanijesu et al.^8^ proposed a temperature-seeking method to investigate the possibility of using pure H_2_ and N_2_ gases to suppress hydrate formation in a subsea gas pipeline. Hydrates of the CH_4_/CO_2_ mixture were formed in a cryogenic sapphire cell to study the interaction of the components. They confirmed that the addition of either gas would either prolong hydrate formation during operation or prevent agglomeration of the formed hydrate. This ensures successful hydrocarbon transport, allowing the industry to operate at any desired pressure while controlling hydrate formation and emissions.

Furthermore, Lowesmith et al.^9^ have developed a mathematical model describing the release of gas when it mixes with air and forms a layer of gas-air mixture in the upper part of the ventilation equipment casing. The proposed model considered both wind and buoyant ventilation resulting from gas accumulation. Hsin-Yi Shih et al.^10^ performed simulations using a three-dimensional compressible k–ε turbulent flow model and an assumed chemical reaction probability density function. They studied the combustion and emission characteristics at a variable hydrogen volume fraction from 0 to 90%. They result of their study show that, as methane is substituted for hydrogen at a fixed fuel injection rate, the flame temperature becomes higher, but the lower fuel consumption and heat input at higher hydrogen substitution percentages cause turbine power shortages. Consequently, to utilize blended fuels at constant fuel consumption, they increased the flame temperature with an increasing percentage of hydrogen, which improved the performance of the gas turbine, but CO_2_ emissions remained major problems. When fixing a certain heat input of the engine with blended fuels, a wider but shorter flame is found with a higher percentage of hydrogen, but a significant increase in CO emissions indicates a decrease in combustion efficiency. For a micro gas turbine engine with fuels blended with hydrogen and methane, further modifications, including fuel injection and cooling strategies, are needed as an alternative. As a result, with the use of mathematical analysis, it will be possible to trace the transient steady-state modes of the methane-hydrogen mixture flow parameters through the compressor station's technological pipelines. The latter will make it possible to control the process pipelines of gas compressor units and centrifugal blowers at the compressor station and, consequently, to predict and prevent sudden changes in the behavior of gas flows (methane and hydrogen), which can lead to terrible accidents at compressor stations.

**Figure 1:**
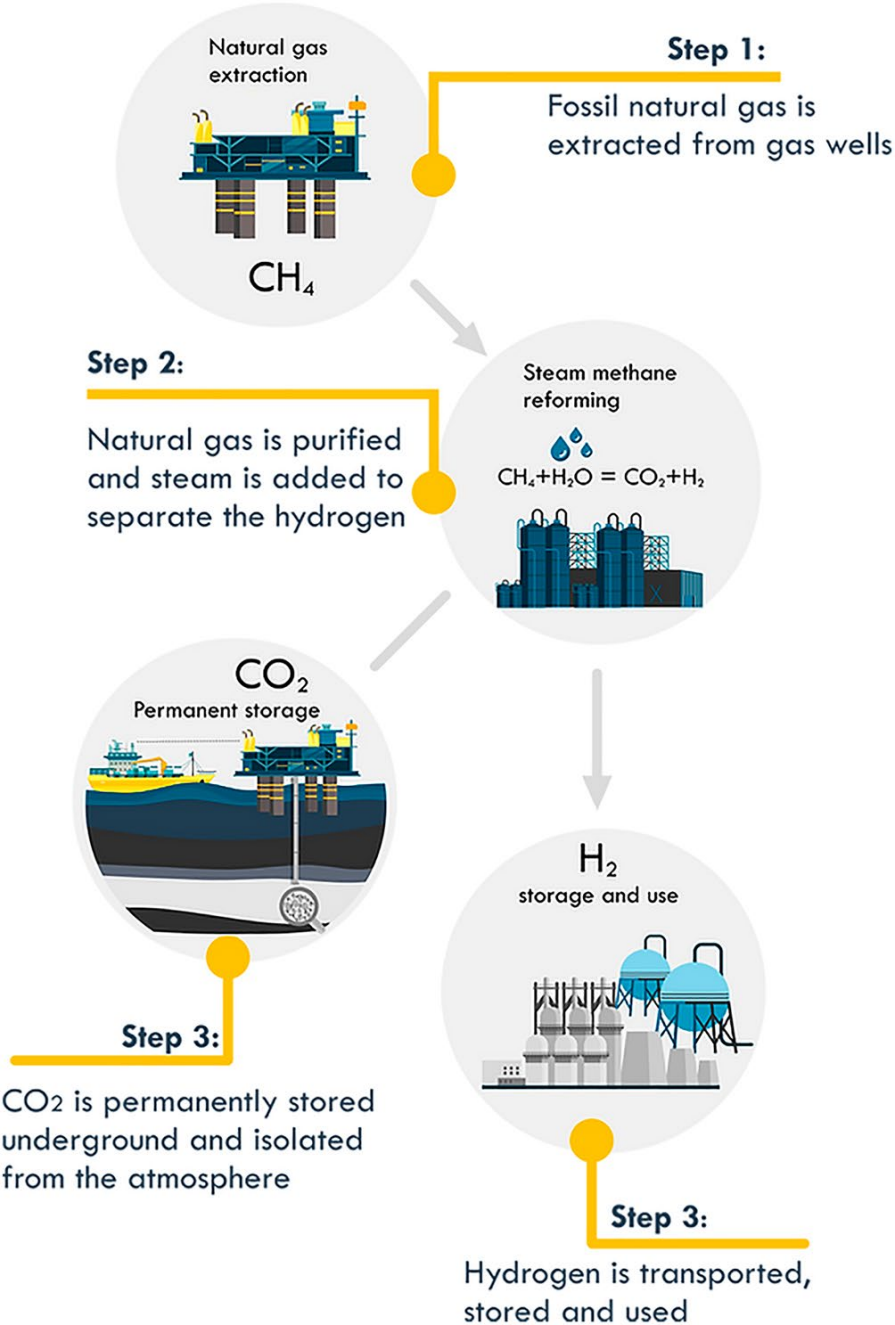
Stages of H_2_/CO_2_ production and storage in the oil and gas industry.

The field of gas flow modeling has garnered growing attention from both engineers and researchers. The reason is that the methane-hydrogen mixture is a promising energy carrier, and its application is rapidly increasing with the development of technical equipment and the demand for hydrogen as an energy carrier^5,6^. From the perspective of basic science, the choice of mixing hydrogen with natural gas and further transporting it through existing gas pipelines^7,8^ makes the control of the transient parameters of the gas flow more interesting to control CO_2_ emissions when using this type of fuel as an energy carrier at all stages of production and storage (Fig. 1).

In fact, for the delivery of gaseous hydrogen to the end users (which are petrochemical plants and industries that use hydrogen as an integral element in the production of end products), methane-hydrogen mixtures can be provided^11–13^ with continuous delivery methods. If existing pipeline systems are utilized^3,14^, transporting methane-hydrogen mixtures through trunk pipelines is an efficient and economical choice. Consequently, injection and sequential mixing of hydrogen with natural gas have been proposed as economical means of hydrogen transport in several studies. Thus, the analysis of steady-state and transient flows of hydrogen mixtures with natural gas is a key point for determining permissible concentrations of hydrogen in natural gas to ensure safe gas mixture transportation through the compressor station process pipeline system^15,16^.

The effect of hydrogen on the pressure drop in pipelines was calculated by Schouten et al.^17^. Moreover, In Brown et al.^18^, analyzed the construction costs of gas pipelines and studied the effect of hydrogen on the global cost. Then, Fekete et al., 2015; Raab et al., 2021 in their study^19,20^ described the calculation of the economic cost of gas pipelines. The cost depends on the diameter of the pipes, and the cost of a hydrogen pipeline can be 50–80% higher than that of a gas pipeline of the same size. They inferred that regional transport costs can be five times higher than those of natural gas, primarily due to the lower volumetric energy density of hydrogen.

It is worth noting that upgrading compressor stations to H_2_ inevitably generates CO_2_ as a by-product. In a low-carbon society, the storage or reuse, where possible, of captured CO_2_ will be of fundamental importance^21,22^. The CO_2_ produced from CH_4_/H_2_ processing can either be transported by pipeline and stored in underground geological formations (i.e., in a sequestration scenario) or converted into a higher-energy synthetic gas through an energy-to-gas conversion process (i.e., using electrolysis and methanation plants)^23,24^. High-temperature electrolysis processes can convert surplus electricity from intermittent, large-scale renewable power plants into synthetic hydrocarbon fuel gases^25,26^. If low levels of hydrogen (up to 10%) should not be critical for pipework, compressors, metering, calculation equipment, and industrial equipment (e.g., gas turbines, gas engines, and compressed natural gas (CNG) fueling stations) (if the Wobbe index is within the required range), there are sensitive components that can be damaged by the presence of hydrogen^27^. There are proposals to inject hydrogen (H_2_) from renewable sources into the natural gas grid. This measure would utilize the very large transport and storage capacity of existing infrastructure, especially high-pressure pipelines, for indirect transport and storage of electricity. Korb et al.^28^ show that the effect of adding hydrogen to natural gas on the operating range, emissions, and efficiency of engines running on depleted gas mixtures is investigated from an experimental point of view. One of the main factors in determining the limit of hydrogen injection into the natural gas network is the behavior of devices or energy conversion technologies in a hydrogen-rich atmosphere, such as engines. Some results are related to gas quality assurance, which includes changes in calorific value, Wobbe index, and mixture density. Safety aspects, strictly related to the amount of hydrogen mixed with the natural gas stream, are decisive points for establishing the actual grid capacity that exceeds the requirements determined by quality issues. Recent interest has been devoted to the analysis of the transient behavior of gas pipelines. In another study Gato and Henriques^29^ provided a numerical simulation of dynamic gas flow in high-pressure pipelines, which is then applied to understand pressure fluctuations due to the rapid closure of a downstream shut-off valve by solving the conservation equations for one-dimensional compressible flow using the discontinuous Runge–Kutta Galerkin method with a third-order approximation in space and time.

The other studies proposed boundary conditions using a new weak formulation based on characteristic variables and studied the occurrence of pressure fluctuations in gas pipelines due to a compression wave arising from the rapid closure of downstream shut-off valves. Also, they analyzed the effect of partial reflection of pressure waves at the transition between pipes of different cross-sectional areas. Considering the modeling of a complete gas network, several models can be found in the literature^30,31^. For example, Malke et al. showed the mathematical optimization of a non-stationary gas network, including the application of an annealing simulation algorithm. They presented how difficult the problem of unsteady gas network optimization is compared to stationary applications. In further studies^32,33^ a non-isothermal dynamic model of a gas transmission network with special attention to mathematical methods capable of solving discontinuities in possible operating states was investigated. In their work, a non-isothermal model of natural gas in pipelines, including mass, momentum, and energy balance equations, is used as a model equation for the modeling and condition assessment of gas pipeline systems. It is shown that the differential equations describing the dynamic behavior of a high-pressure and long-range gas transmission network can be efficiently solved using the orthogonal collocation method. An algorithm for handling discontinuities arising in the dynamic model of the gas transmission network is proposed.

Roger and Conrado^34^ considered in detail the optimization problems applicable to natural gas networks. Several issues are considered, including line packing management, and associated short-term storage, gas quality assurance, and fuel cost minimization at the compressor station. However, the paper is mainly based on an operations research perspective and does not include any review related to the behavior of natural gas networks in low-carbon energy scenarios under CO_2_ emissions^35,36^. Conversely, the analysis of gas pooling problems in natural gas networks could be a generalization of quality assurance for alternative fuel injection into natural gas networks. Only a limited number of papers have been published that include multiple components and gas networks of variable composition. These models appear in some cases for networks with simple topology or are based on a hypothesis that may be restrictive (e.g., isothermal model). Tabkhi et al.^37^ proposed several correlations for expressing the compressibility coefficient in gas flow mathematical modeling in pipelines. The numerical model was solved using a semi-implicit finite volume method to track the transient behavior of the gas^38,39^. By analyzing the other studies^33–35^, several divergent approaches to numerical modeling of steady-state and transient gas flow in pipelines have been proposed. These approaches were developed by computational software as Aspen Hysys [aspenONE Suite v14 (40.0.0.359)], MATLAB [R2024a version 24.1.0.2537033], and Ansys [v2024 R1].

Hydrogen can be transported through an existing gas pipeline either homogeneously or by injection of pure hydrogen into the natural gas stream^40–42^, which is then mixed, and as a result, hydrogen regasification units from natural gas need to be applied. In the study by Weiner^43^, he investigated the impact of hydrogen injection into the gas pipeline system using the method of gas characteristics and gas compressibility coefficients at various hydrogen fractions^44–46^. Many studies devoted to the influence of a fixed amount of hydrogen on the parameters of the gas mixture flow^47–49^, mathematical models of binary gas mixtures^50,51^, momentum, and continuity equations have been analyzed^52^. Thus, in the other studies^53,54^, the authors presented a non-isothermal model of gas flow, which was compared to the isothermal model, through which they proved its applicability in modeling the flow process of methane-hydrogen mixtures over long distances. This description facilitates a physical comprehension of the methane-hydrogen mixture transportation process within the compressor shop system, including potential variations in the operation of gas pumping equipment.

Another issue that should not be ignored is the CO_2_ emissions from gas compressor operations. Existing H_2_ compressors are expensive because the overall compression ratio is very high and, in part, because they require stainless steel construction to avoid metal saturation of hydrogen and the hydrogenation and deterioration of the compressor steel material^55,56^, as well as to absorb CO_2_ in the presence of water vapor. This problem is widely presented in such studies as^57–62^. For example, Vanchugov et al.^57^ analyzed and assessed the underground space for the utilization of carbon dioxide, including after emissions from oil refineries because of hydrogen production. Shirizadeh et al.^58^ modeled the economic path of the energy transition and show that while renewable energy is a key driver of climate neutrality, the role of natural gas is highly dependent on action to reduce associated CO_2_ and methane emissions. Moreover, clean hydrogen (produced mainly from renewable energy sources) can replace natural gas in a large part of its end use, meeting almost a quarter of the final energy demand in climate-neutral Europe. Bistline and Young^59^ assessed the potential role for natural gas and carbon removal in deeply decarbonized electricity systems in the U.S. and evaluated the robustness of these insights to key technology and policy assumptions. They quoted that “We find that natural gas-fired generation can lower the cost of electric sector decarbonization, a result that is robust to a range of sensitivities when carbon removal is allowed under policy”. Wei Liu et al.^60^ describes various methods for producing green hydrogen, which provides methods for calculating greenhouse gas emissions from different hydrogen production pathways. They discuss the world's largest green hydrogen standardization initiative, analyzes the key factors of the global green hydrogen standard, and explain about how to establish quantitative standards and evaluation systems for low-carbon hydrogen, clean hydrogen, and renewable hydrogen using the method in China. Moreover, Frank Markert et al.^61^ compare a range of EOS to predict hydrogen properties typical of different storage types. In their study, tank dynamics are simulated using simplified design and CFD models to evaluate the performance of different EOS for predicting tank pressure, temperature, mass flow, and jet flame length. The selected EOS and selected specific heat correlation are shown to be important for accurately modeling hydrogen evolution at low temperatures that impact air emissions. On the other hand, Zhiyong Li et al.^62^ examined the consequences of accidental emissions from Cryo-compressed hydrogen, LNG, and liquefied natural gas storage facilities for automotive applications. They assessed both non-flammable and flammable effects, including exposure to cold, heat, overpressure, and missiles. The results show that cryo-compressed hydrogen emissions always lead to the worst effects for non-flammable effects, and for flammable effects, the worst effects depend on the ignition time. The longest deadly and dangerous distances are associated with cryo-compressed hydrogen emissions, not CNG and LNG emissions. From a net loss perspective, CNG storage is safer than LNG storage in the event of a catastrophic failure but more dangerous in the event of a leak.

As can be seen from the analysis, the issue of CO_2_ emissions during the transport of a methane-hydrogen mixture remains open, and the topic is interesting from the point of view of the influence of these emissions on the operation of compressor station equipment during gas transportation.

This study is devoted to the development of a mathematical model capable of describing the physical processes of CH_4_/H_2_ transport by pipeline pipework at a compressor station, considering the operation of minimum CO_2_ emissions due to capture technology in a gas turbine unit. In addition, the developed model can consider a gas mixture of different compositions, including the following main compounds: Methane, Ethane, Propane, Butane, Carbon Dioxide, Nitrogen, Carbon Monoxide, and Hydrogen. In general, the developed model is a tool of the system of nonlinear algebraic equations for controlling parameters of safety control of technological modes of CH_4_/H_2_ mixture transportation, as the compression power is a strong function of the thermodynamic process and is determined not only by the compressor efficiency. To optimize heat integration, compression systems should be integrated with both the power unit and CO_2_ capture units. A two-stage CH_4_/H_2_ mixture combustion technology is proposed for high operating efficiency and reduced operating costs in carbon dioxide capture and sequestration systems.

## Methodology

### Description of the model

**Figure 2:**
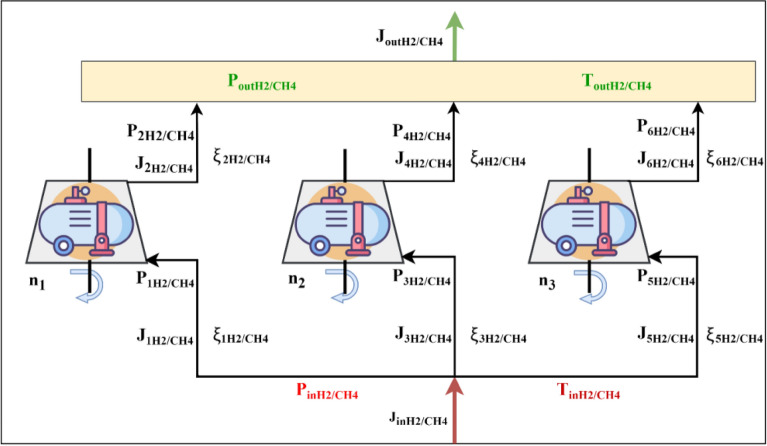
Schematic of the pipeline system under consideration for studying the effect of hydrogen injection on a compressor station.

The CH_4_/H_2_ transportation problem model is general enough to consider various equipment operating modes. As mentioned above, this study examines the impact of CH_4_/H_2_ mixtures in the operation of a compressor station and centrifugal blowers on CO_2_ emissions. The pressure drop in the gas pipeline is an essential parameter for determining the required compression power for the gas, which is described by the differential pulse balance equation. The interaction between the boundary layer of liquid and the inner surface of the pipe results in energy dissipation, which therefore causes a reduction in gas pressure. The fundamental mathematical modeling of the key network elements involves the use of material balance and momentum conservation equations, along with other essential equations. The necessary equations in the gas transmission network system have been developed to determine conditions such as pressure and flow. The balance of moments for one pipeline is formulated in relation to the compressor and its impact on optimizing the operating conditions of the gas pipeline system. Various connections between elementary sections of the network are determined using incidence matrices (Fig. 2). Compressor stations compensate for pressure drops due to friction in pipelines, fittings, and other components, as well as due to elevation changes.

In pipeline networks, compressor stations consume a small share of transported gas^63^. The relationship between the suction and discharge pressures of a centrifugal compressor and the power transferred to the gas is represented by determining the isentropic height of the compressed fluid^64^. This study assumes that the compressor performance, represented by classical characteristic curves, is compatible with the case of transporting a CH_4_/H_2_ mixture.

The normalized parameters P_2H2/CH4_, J_2H2/CH4_, T_2H2/CH4_ and $\xi$_2H2/CH4_ are used to describe the characteristic curves of the resulting compressors. Thus, the rotation speed of all compressors is between the two boundaries.

The example used as a test bed in this study is a gas turbine plant for natural gas application^65^, which is considered for the case of mixtures of CH_4_/H_2_ (Fig. 2). Hydrogen added to natural gas is considered constant during optimization of the composition of natural gas.

In this figure, technological gas pipelines at the compressor station are represented by conditional lines, and the arrows indicate the direction of the gas mixture, which passes through the outlet manifold, which is depicted as a rectangle, and the compressor shop in the form of a trapezoid depicting centrifugal blowers (Supplementary 1).

This didactic network consists of a system of process pipelines, between which are located three compressors that work to compress the gas mixture and regulate the pressure in the transport system. Each gas turbine unit comprises three centrifugal compressors arranged in parallel, which are connected in parallel by pipelines. As for each compressor unit, there is a gas stream carrying fuel to it; fuel streams, to avoid complexity, are not shown in Fig. 2. For each compressor, this flow originates from the incoming unit (red pointer), following the example of a natural gas compressor station.

### Gas mixture parameters

The physical and chemical properties of hydrogen are quite different from those of natural gas. Table 1 shows some indicative values of the relevant properties for the gas chain from source to end user (some of them will be used in the model development). As a result of these opposing properties, a system designed for natural gas cannot be used without appropriate modifications for pure hydrogen, and vice versa. Even the addition of a certain percentage of hydrogen to natural gas will have a direct effect on combustion properties^66^, diffusion into materials, and the behavior of the gas mixture in the air. These aspects are discussed below.

**Table 1:** Comparison of physical properties of hydrogen and methane as the main component of the transported gas mixture.

No	Physical properties	H_2_	CH_4_	Unit
1	Molecular weight	2.02	16.04	g/mol
2	Critical temperature	33.2	190.65	K
3	Critical pressure	1.315	4.55	MPa
4	Acentric factor	− 0.215	0.008	–
5	Vapour density at normal boiling point	1.34	1.82	kg/m^3^
6	Vapour density at 293 K and 1 bar	0.0838	0.651	kg/m^3^
7	Heat capacity at constant pressure at 25 °C	28.8	35.5	J/mol K
8	Specific heat ratio (*C*_p_/*C*_v_)	1.4	1.31	
9	Lower heating value, weight basis	120	48	MJ/kg
10	Higher heating value, weight basis	142	53	MJ/kg
11	Lower heating value, volume basis at 1 atm	11	35	MJ/m^3^
12	Higher heating value, volume basis at 1 atm	13	39	MJ/m^3^
13	Maximum flame temperature	1800	1495	K
14	Explosive (detonability) limits	18.2–58.9	5.7–14	vol% in air
15	Flammability limits	4.1–74	5.3–15	vol% in air
16	Autoignition temperature in air	844	813	K
17	Dilute gas viscosity at 299 K	9 × 10^−6^	11 × 10^−6^	Pa s
18	Molecular diffusivity in air	6.1 × 10^−5^	1.6 × 10^−5^	m^2^/s

**Figure 3:**
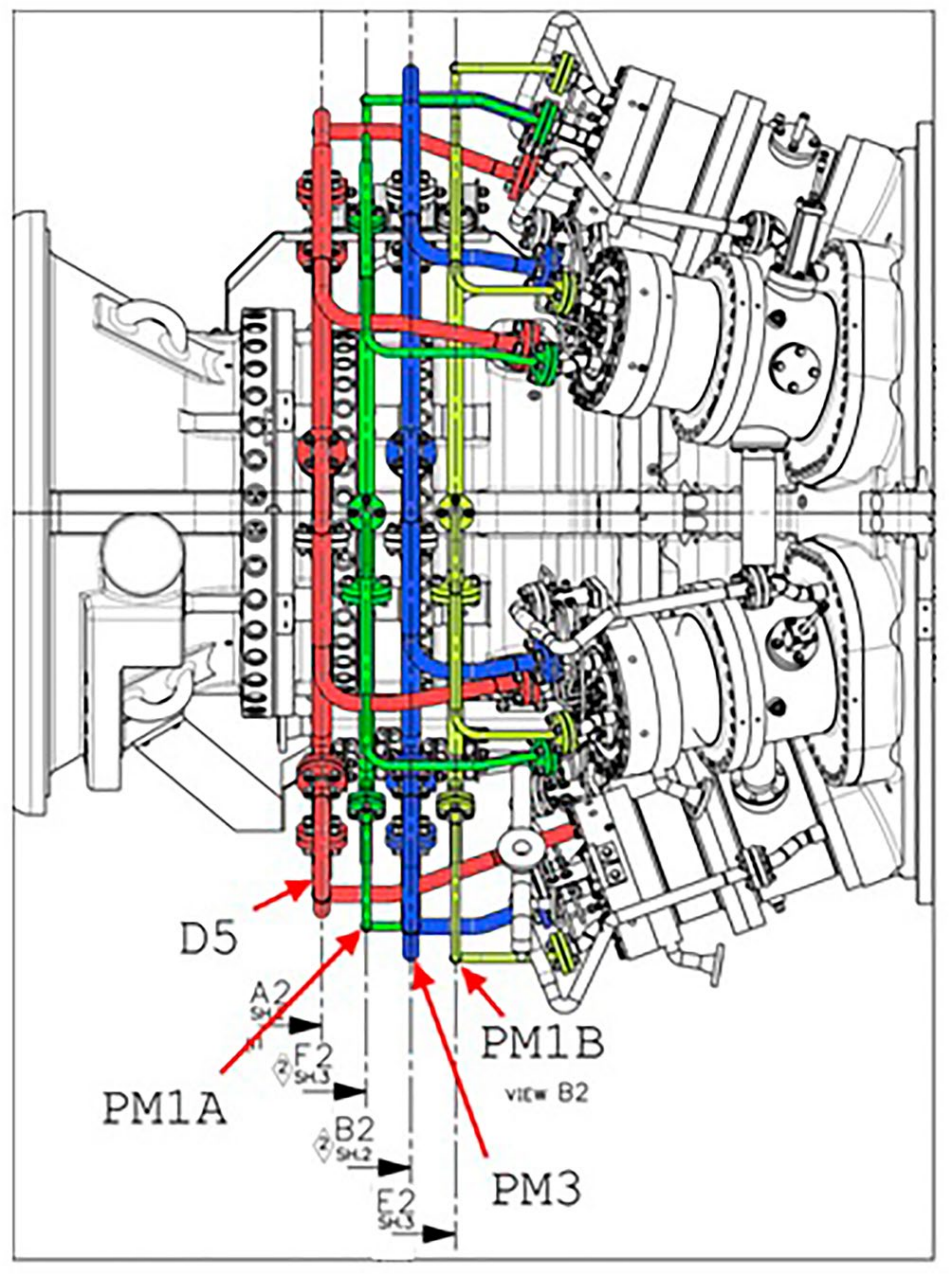
Combustion chamber operation of methane-hydrogen mixture with low emission of CO_2_.

Adding hydrogen to natural gas changes its transportation and calorific properties and affects the safety of fuel delivery in the system, the longevity and reliability of the pipeline, and the operation of all combustion chambers, the mode of which is always the same. It is important to consider the fuel gas supply to the combustion chamber of the gas pipe, particularly when dealing with the H_2_/CH_4_ mixture. The operating mode of all combustion chambers is always the same. The two thickest manifolds are the main ones. These are D5 and PM3. The other two are auxiliary (correction) manifolds. These are PM1A and PM1B. Each combustion chamber has five nozzles. The gas supply to the chambers is more complex than just manifold-to-nozzle. Indeed, only the PM1A and PM1B correction manifolds supply fuel gas to only one "own" injector. The D5 manifold communicates with all five injectors at once, and the PM3 manifold communicates with three injectors. Below is a diagram of the manifold’s communication with the injectors (Fig. 3).

#### Modification of the combustion chamber of a gas turbine plant to accept H_2_

In combustion chambers, the internal energy of fuel during combustion is converted into potential energy for the working body. In modern GTUs, liquid or gaseous fuel is used. Fuel combustion requires an oxidizer, which is air oxygen. High-pressure air enters the combustion chamber after the compressor. The combustion of fuel produces gaseous combustion products of high temperature, which are mixed with additional air. The gas turbine receives the resulting hot gas, known as the working body. According to the proposed solution (Fig. 3), the two thickest collectors are the main collectors. These are labeled as D5 and PM3. The other two manifolds are auxiliary (correction) manifolds; they are PM1A and PM1B. Each combustion chamber has five nozzles. The gas supply to the chambers is more complicated than just connecting the manifold to the nozzles. Indeed, only the PM1A and PM1B correction manifolds supply fuel gas to only one "own" injector. The D5 manifold communicates with all five injectors at once, while the PM3 manifold communicates with three. The scheme of communication between manifolds and injectors is shown in Fig. 3.

A compressor is an integral part of any hydrogen gas storage, transportation, and distribution system. To determine what type of compressor system is needed to do the job, a lot of detailed data must be ascertained. At a minimum, a good knowledge of gas analysis, suction and discharge pressures, suction temperature, and flow rate is required^67^.

To reduce hydrogen leakage in the compressor and the associated risks, compressors must have a proper sealing system installed to ensure safe operation^68^. Explosion-proof components are recommended for all hydrogen compressors. And for those installed in confined spaces, hydrogen concentration sensors are required, as suggested in the example. The compressor outlet pressure varies depending on the type of unit.

Initial data are presented in the value of the coefficient of pressure losses in the gas pipeline process at the compressor station, which is given in the following equations:

1 $${\xi }_{H2i1}=\frac{{\lambda }_{i1}}{{D}_{i1}}+{\left({\xi }_{H2add}\right)}_{i1},$$

And in the downstream process pipelines:

2 $${\xi }_{H2i2}=\frac{{\lambda }_{i2}}{{D}_{i2}}+{\left({\xi }_{H2add}\right)}_{i1},$$

**Figure 4:**
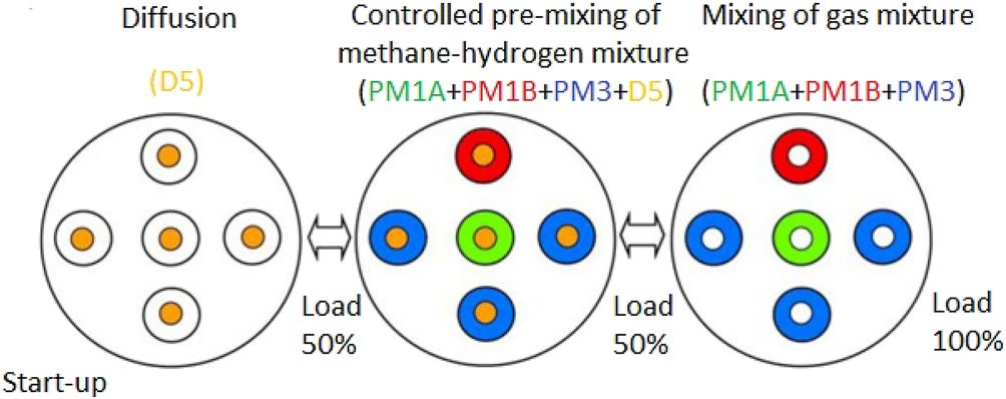
Gas collector (centrifugal blower) for methane-hydrogen mixture.

The choice of compressors largely depends on the initial thermodynamic parameters of the hydrogen entering the pipeline. In all cases, it is assumed that hydrogen is initially generated at 4 MPa and 28 MPa and then compressed to the initial pipeline parameters of 9.8 MPa. Depending on the mass flow rate of hydrogen, three types of compression processes were proposed for the length of the gas pipeline discharging the methane-hydrogen mixture from the centrifugal blower (Fig. 4). Where PM is pre-mixing (pre-mixing of hydrogen and methane), and D is diffusion (diffusion combustion of a gas mixture).

**Figure 5:**
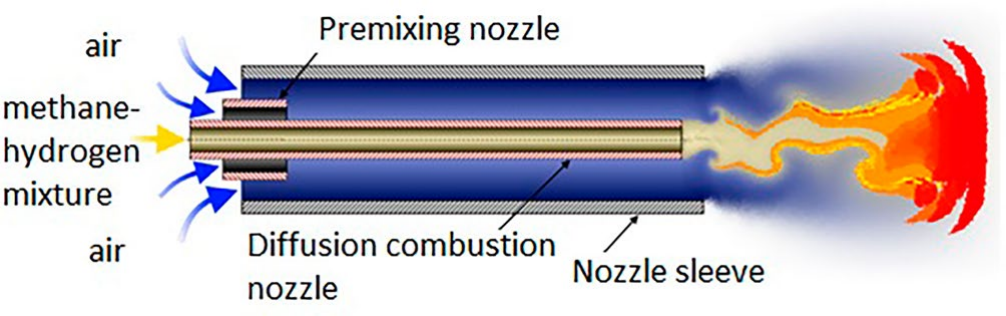
Diffusion combustion.

**Figure 6:**
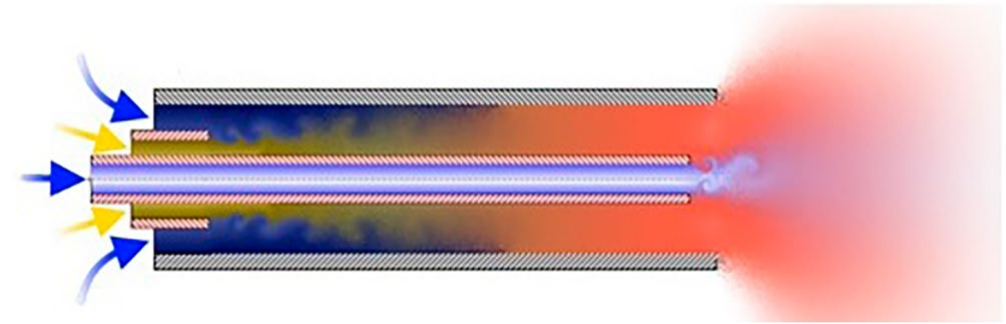
Combustion with pre-mixing of gases.

The numerical value in the term corresponds to the quantity of injectors that are linked to the fuel manifold within a single chamber. D5 manifold supplies fuel gas in the form of a methane-hydrogen mixture to all the combustion chamber nozzles so that it burns before it mixes with air in the diffusion combustion mode, reducing CO_2_ emissions. The remaining manifolds feed the methane-hydrogen mixture to the same nozzles, but in such a way that it is mixed with air before combustion (premixed combustion) (Figs. 5 and 6).

The distinction between the two types of combustion depicted in Figs. 3 and 4 lies in the fact that in Fig. 3, the burning of the methane-hydrogen mixture occurs at the interface with air, whereas in Fig. 4, it takes place uniformly throughout the full volume of the combined mass, referred to as "hydrogen-methane-air". It can be guessed that the second way of combustion is obviously more qualitative and provides lower emissions of harmful gases (mainly CO_2_, CO). However, gas turbine units cannot operate in this mode in the whole range of loads because this combustion is stable only in high loads. Usually, the gas turbine unit is started in diffusion mode, and after gaining 50% of power, it switches to pre-mixing mode (the same way back). The transition occurs almost imperceptibly: collector D5 gradually closes, and collectors PM3, PM1A, and PM1B open by the same amount.

It should be noted that in the diffusion mode, the gas turbine unit can operate in a wide range of loads and usually works like this until the combustion chambers are adjusted. Therefore, for steady-state modeling, it is desirable to have experimental data for all centrifugal blowers. In this case, the methodology uses the nameplate data of the centrifugal blowers, considering the estimated wear characteristics of the equipment.

### Model of methane-hydrogen mixture transportation

In the mathematical model of construction of the compressor unit operation algorithm, objects such as air-cooling units and dust collectors can be included, but it should be considered that the inclusion of these objects will make the calculations complicated, and the inclusion of these models in the description of the general system of compressor shop operation will not affect the stated method of mathematical modeling of the group of gas compressor units. Therefore, in this study, the mathematical description of the air-cooling apparatus's operation and dust collectors is not considered^69^, which does not violate the general condition of the study.

**Figure 7:**
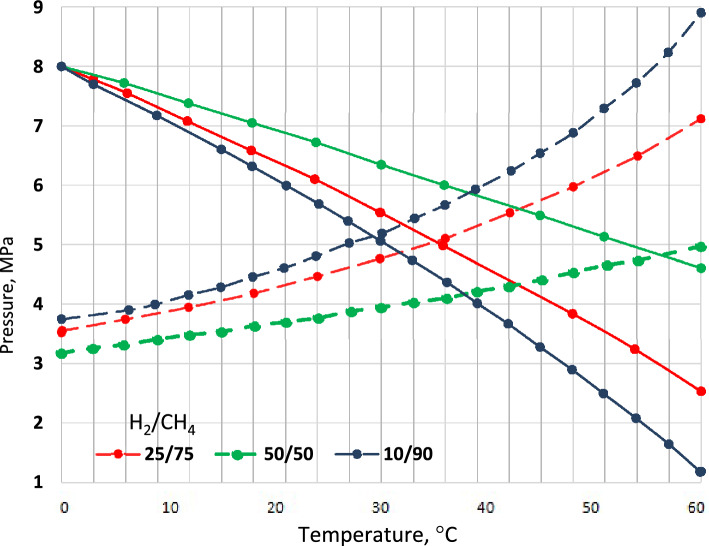
Change in pressure and temperature at the pipeline inlet.

This section models the pipelines to predict their temperature and pressure profiles under various boundary conditions. Consequently, the modeling results are compared with the three approaches found in the literature in terms of predicting pressure and temperature profiles^70^. The results of the comparative evaluations are presented in Fig. 7. These results are obtained for pure hydrogen and methane, considering the pipeline characteristics, shown in Table 2, where the temperature is fixed at 0 to 60 °C under normal environmental conditions of 28 and the total heat transfer coefficient is assumed to be 3.75 W m^−2^ K^−1^.

**Table 2:** Characteristics of the pipeline.

Pipeline parameters	Unit
Length	100, km
Diameter	650, mm
Roughness	10,$\mathrm{\mu m}$
Mass flow rate	62, kg/s
External temperature	28,°C
Heat transfer coefficient [W m^−2^ K^−1^] 3.69	3.7, W m^−2^ K^−1^

In the first case, the model was compared with a steady-state non-isothermal ideal gas flow model, developed under the assumption of ideal heat capacity at constant pressure. Instead, the compressibility coefficient is calculated for each discretization of the pipeline. Figure 7 shows the temperature and pressure profiles calculated using the ideal gas model. Since the steady-state non-isothermal ideal gas flow model is based on ideal gas behavior, it is unable to simulate the Joule-Thompson effect. In fact, the gas reaches the soil temperature after a few meters regardless of the temperature at the inlet section of the pipeline, and no noticeable differences are observed in terms of predicting pressure profiles due to the equality of the mean temperature used for the flow equation.

When modeling the stationary mode of a compressor station when transporting a methane-hydrogen mixture, the following independent variables can be used: the pressure at the inlet of the centrifugal supercharger and the mass flow rate of the methane-hydrogen mixture in the process working pipeline at the compressor station, the compressibility coefficient which is calculated by the Peng-Robinson equation of state for real gas—Robinson to calculate gas equilibrium and thermodynamic parameters.

3 $$p=\frac{RT}{{V}_{m}-b}-\frac{{a}_{\alpha }}{{V}_{m}^{2}+{2bV}_{m}-{b}^{2}},$$

Parameters are described in Eqs. (4)–(5):

4 $$a={\Omega }_{\alpha }\frac{{R}^{2}{T}_{c}^{2}}{{p}_{c}},$$

5 $$b={\Omega }_{b}\frac{R{T}_{c}}{{p}_{c}},$$

6 $${\eta }_{c}={\left[1+{\left(4-\sqrt{8}\right)}^{1/3}+{\left(4+\sqrt{8}\right)}^{1/3}\right],}^{-1}$$

7 $$\alpha ={\left(1+k\left(1-\sqrt{{T}_{r}}\right)\right)}^{2},$$

8 $${T}_{r}=\frac{T}{{T}_{c}},$$

$$k\approx 0.37464+1.5422\omega -0.26992{\omega }^{2},$$

The dimensionless Peng–Robinson parameters In polynomial form:

9 $$A=\frac{\alpha ap}{{R}^{2}{T}^{2}},$$

10 $$B=\frac{bp}{RT},$$

11 $${Z}^{3}-\left(1-B\right){Z}^{2}+\left(A-AB-{3B}^{2}\right)Z-\left(AB-{B}^{2}-{B}^{3}\right)=0$$

In this model, when hydrogen and methane flows are separated at the inlet group of gas pumping units, the process is isothermal. Local resistances in the branching zone of process gas pipelines are neglected to avoid throttling. The pressure distribution in the branching zone is static, i.e., the pressure after the branching zone is equal to the pressure before the branching zone. The temperature of the methane-hydrogen mixture before and after the branching zone will take the form of a constant value. Provided that the mass flow rates before and after separation of the gas mixture flows in the inlet manifold are constant, they will take the form of the below Eqs. (4)–(8):

12 $${\Omega }_{\alpha }=\frac{8+40{\eta }_{c}}{49-37{\eta }_{c}}\approx 0.45724,$$

13 $${\Omega }_{b}=\frac{{\eta }_{c}}{3+{\eta }_{c}}\approx 0.07780,$$

To exclude the occurrence of counter-lows in the gas mixture and to analyze the situation at the shutdown of gas compressor units, inequalities (12) and (13), depending on the set of independent variables, can be applied alternately. In the case of stationary operation of the compressor station pipeline system, the mass flow rates are equal in accordance with the law of conservation of mass. The Peng–Robinson method in its iterative form allows you to calculate the gas compressibility coefficient Z, which must know when selecting equipment for a compressor station:

14 $$Z=\frac{Z}{Z-B}-\frac{AZ}{{Z}^{2}+2BZ-{B}^{2}},$$

The temperature of the methane-hydrogen mixture, which is delivered in sections on the outflow manifold, will have varying limits behind the centrifugal blower, assuming an isothermal operation.

## Model equations

### Dimensionless parameters

**Figure 8:**
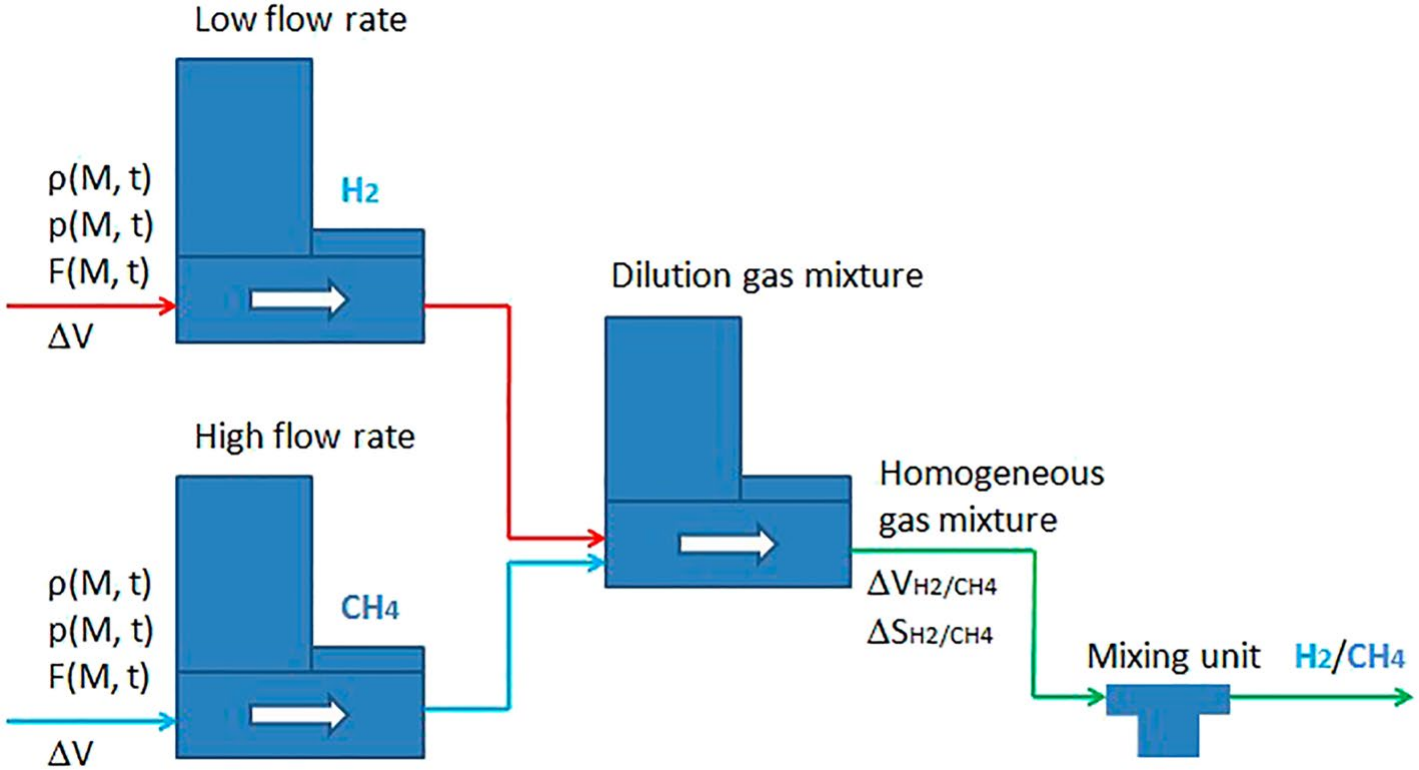
Schematic diagram of the gas mixture flow mixing unit.

In the analytical technology modeling of methane-hydrogen mixture transportation through the compressor shop, we can consider the adiabatic process of mixing gas mixture streams (Fig. 8). In order to create a homogeneous gas mixture, it is not sufficient to simply connect the pipelines supplying hydrogen and natural gas into one single gas mixture. The most characteristic in this respect is the task of adding to the main stream of one gas a small amount of the other. For example, to 75% CH_4_, 25% of H_2_ should be added. In such a situation, there is a high probability of creating an inhomogeneous mixture that varies considerably over time. The homogeneity of the mixture will be affected by the degree of turbulence in the main gas flow, the geometry of the pipeline section where mixing takes place, and the physical properties of the gases, such as density and viscosity. Methane, which constitutes the major part of the gas mixture, is fed perpendicularly through a compression-type connector through a pipeline, while hydrogen is fed parallel to the methane flow. The turbulent, well-mixed and homogeneous CH_4_/H_2_ gas mixture is discharged through the remaining third connector into the combustion chamber. Three flow controllers of appropriate rating and one mixing unit are sufficient.

In this case, it should be considered that in the mixing zone of gas mixture flows, the pressure distribution looks static, i.e., it will be equal to that before the mixing zone (Supplementary 2).

14 $$\frac{\partial u}{\partial x}+\frac{\partial \upsilon }{\partial y}+\frac{\partial \omega }{\partial z}=0$$

15 $$\frac{\partial }{\partial t}\left(pu\right)+\frac{\partial }{\partial x}\left(puu\right)+\frac{\partial }{\partial y}\left(p\upsilon u\right)+\frac{\partial }{\partial z}\left(p\omega u\right)=-\frac{\partial p}{\partial x}+\mu \left(\frac{{\partial }^{2}u}{{\partial x}^{2}}+\frac{{\partial }^{2}u}{{\partial y}^{2}}+\frac{{\partial }^{2}u}{{\partial z}^{2}}\right)$$

16 $$\frac{\partial }{\partial t}\left(p\upsilon \right)+\frac{\partial }{\partial x}\left(pu\upsilon \right)+\frac{\partial }{\partial y}\left(p\upsilon u\right)+\frac{\partial }{\partial z}\left(p\omega \upsilon \right)=-\frac{\partial p}{\partial y}+\mu \left(\frac{{\partial }^{2}\upsilon }{{\partial x}^{2}}+\frac{{\partial }^{2}\upsilon }{{\partial y}^{2}}+\frac{{\partial }^{2}\upsilon }{{\partial z}^{2}}\right)$$

17 $$\frac{\partial }{\partial t}\left(p\omega \right)+\frac{\partial }{\partial x}\left(pu\omega \right)+\frac{\partial }{\partial y}\left(p\omega u\right)+\frac{\partial }{\partial z}\left(p\omega \omega \right)=-\frac{\partial p}{\partial z}+\mu \left(\frac{{\partial }^{2}\omega }{{\partial x}^{2}}+\frac{{\partial }^{2}\omega }{{\partial y}^{2}}+\frac{{\partial }^{2}\omega }{{\partial z}^{2}}\right)$$

The H2/CH4 transfer equations can be represented in the following form:

18 $$\frac{\partial \rho {\dot{M}}_{H2}}{\partial t}+\nabla \cdot \left(\rho \upsilon {\dot{M}}_{H2}\right)=\nabla \cdot \left(\rho {D}_{H2}\nabla {\dot{M}}_{H2}\right)+{m}_{H2}$$

19 $$\frac{\partial \rho {\dot{M}}_{CH4}}{\partial t}+\nabla \cdot \left(\rho \upsilon {\dot{M}}_{CH4}\right)=\nabla \cdot \left(\rho {D}_{CH4}\nabla {\dot{M}}_{CH4}\right)+{m}_{CH4}$$

To describe the mathematical model of compressor shop operation under the condition of methane-hydrogen mixture transportation with given hydrogen parameters can be solved using equations with dimensionless parameters, which play an important role in the research on the study and analysis of fluid behavior. In my case, I used Reynolds number and Reynolds Navier–Stokes (RANS).

The Reynolds number describes the relationship between viscous forces and forces that affect (inertial), these forces can be written as:

20 $$Re=\frac{{F}_{i}}{{F}_{v}}=\frac{{\rho d}^{2}{\upsilon }^{2}}{\mu \upsilon d}=\frac{\rho \upsilon d}{\mu }$$

Using the Reynolds Navier–Stokes (RANS) equations, a time-average statistical method is used in which parameters such as speed, pressure and temperature are decomposed into the sum of a time average and a variation over a specific average.

21 $$\phi =\overline{\phi }+{\phi }^{`}$$

Substituting those variables into Eqs. (14) to (17), the RANS incompressible fluid flow can be written.

22 $$\frac{\partial \overline{u}}{\partial x}+\frac{\partial \overline{\upsilon }}{\partial y}+\frac{\partial \overline{\omega }}{\partial z}=0$$

23 $$\frac{\partial \rho \overline{u}}{\partial t}+\overline{u}\frac{\partial \rho \overline{u}}{\partial x}+\overline{\upsilon }\frac{\partial \rho \overline{u}}{\partial y}\overline{\omega }\frac{\partial \rho \overline{u}}{\partial z}=-\frac{\partial \overline{p}}{\partial x}+\mu \left[\frac{\partial }{\partial x}\right.\left(\frac{\partial \overline{u}}{\partial x}-\overline{u}\right)+\frac{\partial }{\partial y}\left(\frac{\partial \overline{u}}{\partial y}-\overline{u}\right)+\frac{\partial }{\partial z}\left.\left(\frac{\partial \overline{u}}{\partial z}-\overline{u}\overline{\omega }\right)\right]$$

24 $$\frac{\partial \rho \overline{\upsilon }}{\partial t}+\overline{u}\frac{\partial \rho \overline{\upsilon }}{\partial x}+\overline{\upsilon }\frac{\partial \rho \overline{\upsilon }}{\partial y}\overline{\omega }\frac{\partial \rho \overline{\upsilon }}{\partial z}=-\frac{\partial \overline{p}}{\partial x}+\mu \left[\frac{\partial }{\partial x}\right.\left(\frac{\partial \overline{\upsilon }}{\partial x}-\overline{\upsilon }\right)+\frac{\partial }{\partial y}\left(\frac{\partial \overline{\upsilon }}{\partial y}-\overline{\upsilon }\right)+\frac{\partial }{\partial z}\left.\left(\frac{\partial \overline{\upsilon }}{\partial z}-\overline{\upsilon } \overline{\omega }\right)\right]$$

25 $$\frac{\partial \rho \overline{\omega }}{\partial t}+\overline{u}\frac{\partial \rho \overline{\omega }}{\partial x}+\overline{\upsilon }\frac{\partial \rho \overline{\omega }}{\partial y}\overline{\omega }\frac{\partial \rho \overline{\omega \upsilon }}{\partial z}=-\frac{\partial \overline{p}}{\partial x}+\mu \left[\frac{\partial }{\partial x}\right.\left(\frac{\partial \overline{\omega }}{\partial x}-\overline{\omega }\right)+\frac{\partial }{\partial y}\left(\frac{\partial \omega }{\partial y}-\overline{\omega }\right)+\frac{\partial }{\partial z}\left.\left(\frac{\partial \overline{\omega }}{\partial z}-\overline{\upsilon } \overline{\omega }\right)\right]$$

As can be seen from the equations in (23–25), there are six new unknowns that physically represent the transfer of momentum between the turbulent field and the mean field. These terms form the turbulent stress tensor or Reynolds stress tensor $\left({\tau }^{R}=-\rho {\overline{\upsilon }}_{i}^{`}{\overline{\upsilon }}_{j}^{`}\right)$: $${\tau }^{R}=-\rho \left[\begin{array}{c}\overline{u}`\overline{u}` \overline{u}`\overline{\upsilon }` \overline{u}`\overline{\omega }`\\ \overline{\upsilon }`\overline{u}` \overline{\upsilon }`\overline{\upsilon }` \overline{\upsilon }`\overline{\omega }`\\ \overline{\omega }`\overline{u}` \overline{\omega }`\overline{\upsilon }` \overline{\omega }`\overline{\omega }`\end{array}\right]$$

The appearance of the Reynolds tensor terms in the equations with (23–25) makes the system of equations open, which means that there are more unknowns in the RANS equations than in the equations. To close the system, a turbulence model is needed^71^. The presented substitution process is also performed for the hydrogen and natural gas particle transport equations, therefore substituting (21) into Eqs. (18) and (19) to obtain transport equations capable of simulating turbulent flow to describe the absence of countercurrents in the branch process pipelines connecting a group of gas pumping units.

### Construction and numerical analysis of mathematical models

In many problems of gas dynamics, it is possible to disregard the molecular structure of the gas and consider the gas mixture of hydrogen and natural gas as a continuous medium. When talking about infinitesimal small elements of the volume of a gas mixture, it is implied that the volume is small compared to the characteristic size of the system but contains a very large number of molecules^72^. Similarly, when one speaks of the motion of hydrogen and methane gas particles, one does not mean the motion of a single gas molecule, but the displacement of an element of the gas volume containing many molecules, in gas dynamics this is considered as a point.

Suppose the gas mixture is travelling at a velocity $v \left(M,t\right)=v(x,y,z,t)$, projections, where on the coordinate axes denote ${v}_{x},{v}_{y},{v}_{z}.$ Note that, $v \left(M,t\right)$ is the velocity of the gas mixture at a given point $v (x,y,z)$, of space at time t, that is, it refers to specific points in space, not to specific particles of gas moving in space.

Let us also introduce the density of hydrogen and natural gas $\rho \left(M,t\right)$, the pressure $p\left(M,t\right)$ and the density of external acting forces *F(M,t),* calculated per unit mass. In this way of description, the problem is said to be considered in Euler coordinates. First, let us obtain the equation of motion of the gas mixture. Let us denote by $\Delta {V}_{H2/CH4}$ some volume of hydrogen in natural gas bounded by the surface $\Delta {S}_{H2/CH4}$. The equidistance of the pressure forces applied to the surface $\Delta S$, is equal to − $\underset{{S}_{H2/CH4}}{\overset{\Delta {V}_{H2/CH4}}{\int }}pnd\sigma$, where *n* is the unit vector of the external normal and the surface $\Delta S$.

To transform this integral we will use the Ostrogradsky formula, which can characterize the processes occurring in technological pipelines transporting methane-hydrogen mixture:

26 $${\int }_{\Delta {S}_{H2/CH4}}^{\Delta {V}_{H2/CH4}}pcos\left(n,x\right)d\sigma ={\int }_{\Delta {S}_{H2/CH4}}^{\Delta {V}_{H2/CH4}}\frac{\partial p}{\partial x}dV,$$

27 $${\int }_{\Delta {S}_{H2/CH4}}^{\Delta {V}_{H2/CH4}}pcos\left(n,y\right)d\sigma ={\int }_{\Delta {S}_{H2/CH4}}^{\Delta {V}_{H2/CH4}}\frac{\partial p}{\partial y}dV,$$

28 $${\int }_{\Delta {S}_{H2/CH4}}^{\Delta {V}_{H2/CH4}}pcos\left(n,z\right)d\sigma ={\int }_{\Delta {S}_{H2/CH4}}^{\Delta {V}_{H2/CH4}}\frac{\partial p}{\partial z}dV.$$

$$\mathrm{As }pn=ip\mathrm{cos }\left(nx\right)+jp\mathrm{cos }\left(ny\right)+kp{\text{cos}}(nz),$$

where, $i,j,k is$ unit vectors of the orthonormalised basis, multiplying formula (26) by *i*, formula (27) by *j*, formula (28) by *k*, we obtain:

29 $$\underset{{\Delta S}_{H2/CH4}}{\overset{\Delta {V}_{H2/CH4}}{\int }}p,n,d,\sigma =\underset{{\Delta S}_{H2/CH4}}{\overset{\Delta {V}_{H2/CH4}}{\int }}{p}_{i},{n}_{i},{d}_{i},{\sigma }_{i}gradpdV.$$

Considering the last formula, the equation of motion for the volume of methane–hydrogen mixture $\Delta {V}_{H2/CH4}$ in the integral form will be as follows:

30 $${\int }_{\Delta {V}_{H2/CH4}}\frac{\partial v}{\partial t}dV=-\underset{\Delta {V}_{H2/CH4}}{\int }{p}_{i},{n}_{i},{d}_{i},{\sigma }_{i}gradpdV+\underset{{\Delta V}_{H2/CH4}}{\int }\rho FdV,$$

calculating the acceleration $\frac{\partial v}{\partial t}$ of some particles of a gas mixture, it is necessary to consider the displacement of a particle of each component. The trajectories of individual particles of a gas mixture are defined by Eqs:

31 $$\frac{dx}{dt}={v}_{x}, \frac{dy}{dt}={v}_{y}, \frac{dz}{dt}={v}_{z},$$

where;

32 $$\frac{dv}{dt}=\frac{\partial v}{dt}+\frac{\partial v}{\partial x}\frac{dx}{dt}+\frac{\partial v}{\partial y}\frac{dy}{dt}+\frac{\partial v}{\partial z}\frac{dz}{dt}=\frac{\partial v}{dt}+\frac{\partial v}{\partial x}{v}_{x}+\frac{\partial v}{\partial y}{v}_{y}++\frac{\partial v}{\partial z}{v}_{z}=\frac{\partial v}{\partial t}+\left(v\nabla \right)v,$$

here the operator $v\nabla$ is defined as follows:

33 $$v\nabla ={v}_{x}\frac{\partial }{\partial x}+{v}_{y}\frac{\partial }{\partial y}+{v}_{z}\frac{\partial }{\partial z}.$$

Considering that the functions included in the equation of motion for the volume of gas in integral form are smooth enough, let us carry out our standard procedure: applying the mean value formula and going to the limit, shrinking the volume of methane-hydrogen mixture $\Delta {V}_{H2/CH4}$ to a point, we obtain the equation of motion of the gas mixture in Euler form:

34 $${v}_{t}+\left(v\nabla \right)v=-\frac{1}{\rho }gradp+F.$$

Let us now derive the continuity equation expressing the law of conservation of matter. Let the separated volume $\Delta {V}_{H2/CH4}$ have no gas sources and sinks. Then the change per unit time of the quantity of the gas mixture enclosed inside the volume $\Delta {V}_{H2/CH4}$, is equal to the gas flow across the boundary:

35 $$\frac{d}{dt}{\int }_{\Delta {V}_{H2/CH4}}\rho dv=-{\int }_{\Delta {S}_{H2/CH4}}\rho vnd\sigma .$$

The transform the first part of formula (35) by Ostrogradsky's formula:

36 $${\int }_{\Delta {S}_{H2/CH4}}\rho vnd\sigma ={\int }_{\Delta {V}_{H2/CH4}}div\left(\rho v\right)dV,$$

We obtain:

37 $${\int }_{\Delta {V}_{H2/CH4}}\left\{\frac{\partial \rho }{\partial t}+div\right.\left.(\rho v)\right\}dV=0.$$

Applying the mean value formula and going to the limit, we obtain the equation of continuity of gas mixture flow through the pipeline system:

38 $$\frac{\partial \rho }{\partial t}+div\left(\rho v\right)=0.$$

To the obtained equation of motion of the gas mixture and the equation of continuity of flow it is necessary to add the thermodynamic equation of state, which can be written in the form:

39 $$p=C\left(\rho \right),$$

where, C is a given function.

The result is a system of five scalar equations with respect to five unknown functions *vx, vy, vz,p* and *ρ*:

40 $$\frac{\partial v}{\partial t}+\left(v\nabla \right)v=F-\frac{1}{\rho }gradp,$$

41 $$\frac{\partial \rho }{\partial t}+div\left(\rho v\right)=0,$$

42 $$p=C\left(\rho \right).$$

Thus, have a closed system of equations of gas dynamics.

In many problems of gas dynamics, it is possible to disregard the molecular structure of the gas and consider the gas mixture of hydrogen and natural gas as a continuous medium^73^. When talking about infinitesimal small elements of the volume of a gas mixture, it is implied that the volume is small compared to the characteristic size of the system but contains a very large number of molecules. Similarly, when one speaks of the motion of hydrogen and methane gas particles, one does not mean the motion of a single gas molecule, but the displacement of an element of the gas volume containing many molecules, in gas dynamics this is considered as a point. Thus, we have a closed system of equations of gas dynamics.

Due to the smallness of oscillations in a sound wave, the velocity *v* in it is small, so that in the equation $\frac{\partial v}{\partial t}+\left(v\nabla \right)v=F-\frac{1}{\rho }gradp,$ we can neglect the second-order terms of the form ${v}_{x}\frac{\partial {v}_{x}}{{\partial }_{x}}$ and etc. For the same reason, the relative density and pressure changes of hydrogen and natural gas between the layers are also small.

Let's suppose $$\overline{p} \left(M,t\right)={p}_{0}\left(M\right)+\overline{p}, \overline{\rho } \left(M,t\right)={\rho }_{0}\left(M\right)+\overline{\rho }$$

where ${p}_{0}\left(M\right) and {\rho }_{0}\left(M\right)$ is equilibrium values of gas pressure and density for the two mixtures; $\overline{p} \left(M,t\right) and \overline{\rho } \left(M,t\right)$ re the changes in the sound wave, that $\overline{p} <<{p}_{0, }\overline{\rho }<<{\rho }_{0}$; $\overline{p} \left(M,t\right)$ is the sound pressure.

Neglecting the second order terms in the system, we obtain a linearized system. We decompose the function $C (\rho )$ into a series of powers of $\rho$ and consider the first order terms. As a result, we obtain:

43 $${p}_{0}+\overline{p}=C\left({\rho }_{0}\right)+{C}^{`}\left({\rho }_{0}\right)\overline{\rho }, and so as {p}_{0}=C\left({\rho }_{0}\right),\Rrightarrow \overline{p}={C}^{`}\left({\rho }_{0}\right)\overline{\rho }$$

Thus, the closed system of small acoustic oscillations in a continuous medium will have the form:

44 $${\rho }_{0}\frac{\partial v}{\partial t}=-grad\overline{p}+{\rho }_{0}F,$$

45 $$\frac{\partial \overline{\rho }}{\partial t}+div\left({\rho }_{0}v\right)=0,$$

46 $$\overline{p}={C}^{`}\left({\rho }_{0}\right)\overline{\rho }.$$

Now we obtain the equation of the relative function $\overline{\rho } \left(M,t\right)$. Let's differentiate the equation $\frac{\partial \overline{\rho }}{\partial t}+div\left({\rho }_{0}v\right)=0,$ to *t* and let`s get:

47 $$\overline{{\rho }_{u}}+div\left({\rho }_{0}v\right)=0,$$

And let's use the operator *div* on the equation ${\rho }_{0}\frac{\partial v}{\partial t}=-grad\overline{p}+{\rho }_{0}F,$ let`s get:

48 $$div\left({\rho }_{0}{v}_{t}\right)=-divgrad\overline{p}+div\left({\rho }_{0}F\right).$$

In the linear approximation from $\overline{p}={C}^{`}\left({\rho }_{0}\right)\overline{\rho }$ let`s get:

49 $$grad \overline{p}\cong {C}^{`}\left({\rho }_{0}\right)grad \overline{\rho }.$$

Let us denote $k\left(M\right)={C}^{`}\left({\rho }_{0}\right)$ and $f \left(M,t\right)=-div\left({\rho }_{0}F\right).$ Then from the last three equations we obtain the second order equation with respect to the function $\overline{\rho } \left(M,t\right)$ for the oscillation equation in the three-dimensional case:

50 $$\overline{{\rho }_{u}}+div\left(k\left(M\right)grad \overline{\rho }\right)+f\left(M,t\right),$$

In the case of adiabatic process, the equation of gas state has the form:

51 $$p={p}_{0} {\left(\frac{\rho }{{\rho }_{0}}\right)}^{\gamma },$$

where, $\gamma is$ constant, adiabatic exponent $\gamma =\frac{{c}^{p}}{{c}^{v}};$ ${c}^{p}$ is heat capacity at constant pressure; ${c}^{v}$ is heat capacity at constant volume.

In the linear approximation will have:

52 $$p={p}_{0}+\overline{p}={p}_{0}{\left(\frac{\rho }{{\rho }_{0}}\right)}^{\gamma }={p}_{0}{\left(1+\frac{\overline{\rho }}{{\rho }_{0}}\right)}^{\gamma }\cong {p}_{0}\left(1+\gamma \frac{\overline{\rho }}{{\rho }_{0}}\right),$$

53 $$\mathrm{Where }\,\overline{p}=\gamma \frac{\rho }{{\rho }_{0}}\overline{\rho }$$

The compare Eq. (29) with Eq. (46), and obtain:

54 $$k\left(M\right)= \gamma \frac{{p}_{0}(M)}{{\rho }_{0}(M)}$$

Thus, the model of differential equations describing the changes occurring in the process pipeline at the compressor station, which occur because of oscillatory movements of the gas mixture at the sound wave, at each point of which there is a transverse compression and rarefaction of the gas mixture.

In this model, when hydrogen and methane flows are separated at the inlet group of gas pumping units^75^, the process is isothermal^74^. Local resistances in the branching zone of process gas pipelines are neglected to avoid throttling^75^. The pressure distribution in the branching zone is static, i.e., the pressure after the branching zone is equal to the pressure before the branching zone ($${P}_{H2in})$$.The temperature $$({T}_{H2in})$$ of the methane-hydrogen mixture before and after the branching zone will take the form of a constant value^76^.

### Gas mixture diffusion

Let $u (M,t)$ is concentration of a substance at point M at time t. Let us write by diffusion:

55 $$\overrightarrow{\varphi }\left(M,t\right)=-d\left(M\right)\nabla u\left(M,t\right),$$

where, $\overrightarrow{\varphi }$ is flow of gas mixture; *d (M)* is diffusion coefficient.

Then, the change of the quantity of matter in the region $\Delta V$ for time $\Delta t$ will have the form:

56 $$\Delta m=\underset{{\Delta S}_{H2/CH4}}{\overset{{\Delta V}_{H2/CH4}}{\int }}\left[u\left(M,t+\Delta t\right)-u\left(M,t\right)\right]dV=\underset{{\Delta S}_{H2/CH4}}{\overset{{\Delta V}_{H2/CH4}}{\int }}\left[\underset{{\Delta S}_{H2/CH4}}{\overset{{\Delta V}_{H2/CH4}}{\int }}{u}_{t}\left(M,\tau \right)d\tau \right]dV=\underset{t}{\overset{t+\Delta t}{\int }}d\tau \underset{{\Delta S}_\frac{H2}{CH4}}{\overset{{\Delta V}_\frac{H2}{CH4}}{\int }}{u}_{t}\left(M,\tau \right)d\tau dV.$$

In this case, it should be considered that in the mixing zone of gas mixture flows, the pressure distribution looks static, i.e., it will be equal to that before the mixing zone. In this case, taking all simplifications and assumptions of the condition of conservation of the mass flow rate of the gas mixture into account, the expression (57) will have below form:

57 $$\Delta {m}_{1}=\underset{t}{\overset{t+\Delta t}{\int }}d\tau \underset{\delta }{\overset{t+\Delta t}{\int }}\left(\overrightarrow{\varphi }\left(M,t\right),\overrightarrow{n}\right)dS=\underset{t}{\overset{t+\Delta t}{\int }}d\tau \underset{{\Delta V}_{H2/CH4}}{\overset{t+\Delta t}{\int }}div\left(\overrightarrow{\varphi }\left(M,t\right)\right)dV=-\underset{t}{\overset{t+\Delta t}{\int }}dt\underset{{\Delta V}_{H2/CH4}}{\overset{t+\Delta t}{\int }}div\underset{{\Delta V}_{H2/CH4}}{\overset{t+\Delta t}{\int }}div\left(d\left(M\right)\nabla u(M,t\right)dV.$$

If the space $\Delta V$ contain external sources (heat absorbers), then for time $\Delta t$ they can release matter equal to:

58 $${\Delta m}_{2}=\underset{t}{\overset{t+\Delta t}{\int }}d\tau \underset{{\Delta V}_{H2/CH4}}{\overset{t+\Delta t}{\int }}f\left(M,\tau \right)dV,$$

where, $f\left(M,\tau \right)$ is specific power of matter sources, which is determined by the amount of matter emitted by external sources in a unit volume per unit time, which can be written as the law of conservation of matter:

59 $$\Delta m={\Delta m}_{2}-\Delta {m}_{1},$$

In this case, the temperature at the compressor shop outlet manifold is determined by the formula:

60 $$\underset{t}{\overset{t+\Delta t}{\int }}d\tau \underset{{\Delta V}_{H2/CH4}}{\overset{t+\Delta t}{\int }}{u}_{t}\left(M,t\right)dV=\underset{t}{\overset{t+\Delta t}{\int }}dt\underset{{\Delta V}_{H2/CH4}}{\overset{t+\Delta t}{\int }}f\left(M,t\right)dV+\underset{t}{\overset{t+\Delta t}{\int }}d\tau \underset{{\Delta V}_{H2/CH4}}{\overset{t+\Delta t}{\int }}div\left(d\left(M\right)\nabla u(M,t\right)dV.$$

Let's apply the mean value formula and go to the limit, tending to $\Delta V\to 0,\Delta t\to 0:$

61 $${u}_{t}\left(M,t\right)=div\left(d\left(M\right)\nabla u\left(M,t\right)\right)+f\left(M,t\right).$$

**Figure 9:**
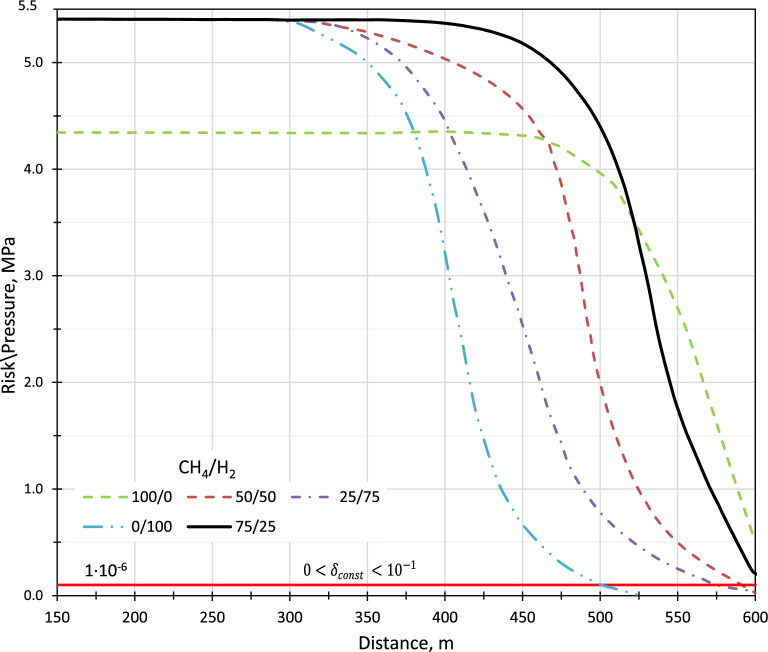
Changes in the mode of methane-hydrogen mixture transportation because of gas mixing.

To build a numerical model for transporting a methane-hydrogen mixture, considering changes in the transportation method, the dependence of influencing factors such as the hydrogen content in natural gas on system failure was calculated (Fig. 9). Thus, the Green's function is formed through a system of Fourier series, which should be supplemented with simple restrictions on the desired variables, considering limiting inequalities. To construct a numerical model for transporting a methane-hydrogen mixture, considering changes in the transportation method, the dependence of the influencing factors of system failure and the pressure drop in steady state caused by friction between the gases and the pipeline wall was established.

It follows that the pressure in all parallel branches of the pipeline system of the considered group of gas compressor units at the compressor station is the same. By analyzing the mathematical model, which is a set of as a Green's function through Fourier series. That describe the parameters of steady-state modes of methane-hydrogen mixture transportation through a group of gas compressor units of a compressor station, equations describe operational, technological and construction features.

If: $d=const\Rightarrow {u}_{t}=d\Delta u+f\left(M,t\right),$ then at stationary diffusion $\left(\frac{\partial }{\partial t}=0\right)$, which is described by the Poisson equation: $\Delta u=-\frac{f(M)}{d}$ changes in the operation of gas compressor units occur under the condition that $f\equiv 0\Rightarrow \Delta u=0.$

Equations show the minimum flow rate value, which corresponds to the maximum pressure value, and vice versa. The constraints’ description on the variables describes the methane-hydrogen’s transport technology as well as the technical condition of the equipment and design features of the pipeline system. Description of values, which consider technical and other characteristics in mathematical modeling of the pipeline system at the compressor station, ensure the condition of preservation of mass flow rates of the gas mixture before and after the conditional point of separation of gas flows.

## Results and discussion

This section presents the results of an optimization problem considering a system of nonlinear algebraic equalities to describe the safety parameters for controlling technological modes of transporting a CH_4_/H_2_ mixture through the technological pipelines of a compressor station. Based on the above formulas, the following methodology is presented for the selection of restrictions on the transport of methane-hydrogen mixtures through the system of technological pipelines at the compressor station:

1. The expressions of initial conditions for all gas pumping units of the modelled group are found, considering the hydrogen concentration: ${\left.u\right|}_{t=0}=\varphi \left(M\right).$
2. The gas pumping unit with the largest area of permissible values and boundary conditions is determined: ${\left.u\right|}_{S}=\mu \left(P,t\right).$
3. Based on claim 2, for the selected gas pumping unit, means that a predetermined concentration of the methane-hydrogen mixture is maintained at the boundary:
  $${\left.\frac{\partial u}{\partial n}\right|}_{{\Delta S}_{H2/CH4}}=v \left(P,t\right).$$
4. The mathematical description of the whole system of equations of the gas pumping system operation will be very labor-intensive from the point of view of the analytical component. Therefore, the mathematical model can be simplified by passing from consideration of the parametric difference equation to consideration of the Green's function through the system of Fourier series as an example for natural gas with hydrogen corrections.

In the presented work several differential equations describing physical processes of different nature have been considered on the example of transportation of methane-hydrogen mixture through process pipelines through the compressor shop. When comparing mass flow rates in each branch of the pipeline system, both indices will have relatively low values. Therefore, the use of quadratic values of mass flow rates in the model is quite justified^77^. The first preliminary optimization task is to minimize the total fuel consumption at the compressor station at a constant pipeline capacity. The pressure is 5 MPa at the inlet, as well as the gas injection point at the nodes with 5.5–6 MPa.

However, in this problem, finding the starting point is a bit difficult because the variables are implicitly related to each other by highly nonlinear constraints. The calculation results are presented in Table 3.

**Table 3:** Optimal values of discharge flow, rotation speed, fuel consumption, isentropic pressure, and isentropic efficiency of a centrifugal supercharger.

Centrifugal supercharger	Unit
Discharge flow (kg/s)	50.67
Rotation speed (s)	287.43
Fuel consumption (kg/s)	0.184
Consumption Rate (%)	0.275
Isentropic head (kJ/kg)	14.72
Isentropic efficiency (%)	75.11

For each centrifugal supercharger, the flow coefficient is defined as the fuel flow rate divided by the input mass flow rate. In this problem, fuel consumption is kept constant in the form of a constraint. The curve in Fig. 9 expresses the optimal values of the consumed share CH_4_/H_2_ in percentage terms depending on the transmitted power at the end points of the network. According to this graph, an increase in energy transmitted through the pipeline leads to an increase in the share of transported gas consumed at compressor stations. It must be said that beyond the top of the curve the optimization procedure ends up with an unfeasible solution.

## Conclusions

This study proposed its own view on the mathematical model of methane and hydrogen transport through an existing gas pipeline in a compressor station system and presented a proposal for improving a centrifugal supercharger to reduce CO_2_ emissions. The main interest of this work is to consider the amount of hydrogen that can be added to a pipeline network traditionally dedicated to transporting natural gas, without any changes to the system. The key to this research is to determine the conditions under which hydrogen can be added to natural gas, and how much hydrogen can be injected into an existing pipeline network^78,79^ while minimizing fuel consumption.

The main hydraulic limiting factor for introducing hydrogen into an existing pipeline is that the specific volume of hydrogen is much greater than the corresponding volume of natural gas, resulting in a severe reduction in pipeline capacity (mass flow) and therefore the energy transferred^80,81^.

Several operational variables were selected as solution variables^82^ for the gas pipeline optimization problem. Optimization procedures^83^ including minimizing fuel consumption, amount of hydrogen added, maximizing power transfer and discharge pressure were performed for various gas mixtures of natural gas and hydrogen^57,84,85^.

The maximum achievable fraction of hydrogen that can be added to natural gas for the example studied is about 25 wt.%. which significantly reduces the transmitted power.

According to this study, adapting existing natural gas transmission networks to hydrogen transportation seems possible until low values are achieved that can be quantified using system optimization tools.

The perspective of this work is now to consider the constraints or safety criteria during the design and operation phase, and to determine the values of CO_2_ emissions resulting from the operation of the compressor shop. In this context, the use of nonlinear algebraic equations and inequalities is, in my opinion, an interesting look at a new opportunity to describe processes in a system.

Solving the system of Green's function through a system of Fourier series showed that the objectives of this study were successfully met. When additional variables, such as the level of CO_2_ emissions produced by the gas turbine and the gas compressor unit, are considered, the existing system of equations can be modified to include both equalities and inequalities. The equations presented by both groups are comparable in terms of physics. In general, the equation model for gas pumping units allows using physical quantities to understand possible changes in the operation of gas pumping equipment during transportation of methane-hydrogen mixture through compressor shop units. The adaptation of the system of equations should be described as an algorithm of a computer programme to fully understand the effect of hydrogen on the operation of the gas pumping unit. The results of calculations using mathematical models coincide based on the condition of mass flow rate conservation through the inlet and outlet process pipelines. Within the framework of realization of mathematical models for gas pumping units operating under the parallel-sequential scheme, the parameter of total fuel gas consumption in the gas turbine unit (or electricity) at use of electric drive is considered. This parameter is determined by summing up the energy consumption at each gas pumping unit and depends on the power of the centrifugal blower.

## Supplementary Information

Supplementary Information.

## Data Availability

The data that support the findings of this study are available on request from the corresponding author. The data are not publicly available due to containing information that could compromise the privacy of research participants.

## List of symbols

$${H}_{2}$$: Hydrogen
${CH}_{4}$: Methan (natural gas)
CO_2_: Carbon dioxide
$${t}_{f}$$: Turbulent flow
$$\left(x, y, z\right)$$: Cartesian components
$\alpha ,\beta$: Injection angle relative to flow direction
$$\epsilon$$: Wall roughness
$k$: Von–Kármán constant
$$\mu$$: Dynamic viscosity
${\mu }_{t}$: Turbulent Eddy viscosity
$$\nu$$: Momentum diffusivity
$\omega$: Rate at which turbulent kinetic energy is converted into thermal energy
$\sigma$: Prandtl numbers(variance)
$$\tau$$: Shear stress
${\overline{\tau }}_{Re}$: Reynolds stress tensor
$\varepsilon$: Average rate of dissipation of turbulent kinetic energy
$c$: Concentration volume fraction (constant)
$$e$$: Relative error
$$TI$$: Turbulence intensity
$$U$$: Mean velocity
$\left(u, v, w\right)$: Velocity Cartesian components
$${u}_{\tau }$$: Friction velocity
$$v$$: Velocity vector
$${y}^{+}$$: Characteristic length
$$`$$: Fluctuation
$$+$$: Wall function
$$A$$: Area
$$D$$: Diameter
$\overline{E}$: Energy transport rate
$F$: External body force
$f$: Moody frictional factor
${f}_{N}$: Numerical friction factor
$$g$$: Gravitational acceleration
${G}_{b}$: Generation of turbulence kinetic energy
${G}_{k}$: Generation of turbulence kinetic energy due to mean velocity gradients
${h}_{L}$: Frictional head loss
$$I$$: Unit tensor
$k$: Turbulence kinetic energy
$${k}_{s}$$: Grain roughness
$$L$$: Length
$m$: Mass flow rate
${m}_{pq}$: Mass transferred from phase *p* to phase *q*
${m}_{qp}$: Mass transferred from phase *q* to phase *p*
$$P$$: Pressure
$$Re$$: Reynolds number
$$S$$: Source term
$$t$$: Time
$$T$$: Temperature
$$u$$: Inlet velocity
$\upsilon$: Average flow velocity
$$V$$: Volume
$$x$$: Displacement
${Y}_{m}$: The contribution of the fluctuating dilatation
${\alpha }_{q}$: Volume fraction of *q*-th fluid
$\rho$: Gas or fluid density
