# Supplementary figures and images for: Analysis of numerical modeling of steady-state modes of methane–hydrogen mixture transportation through a compressor station to reduce CO2 emissions
Source: Sci Rep. 2024 May 8;14:10605. doi: 10.1038/s41598-024-61361-3 (PMC11636934; doi:10.1038/s41598-024-61361-3)

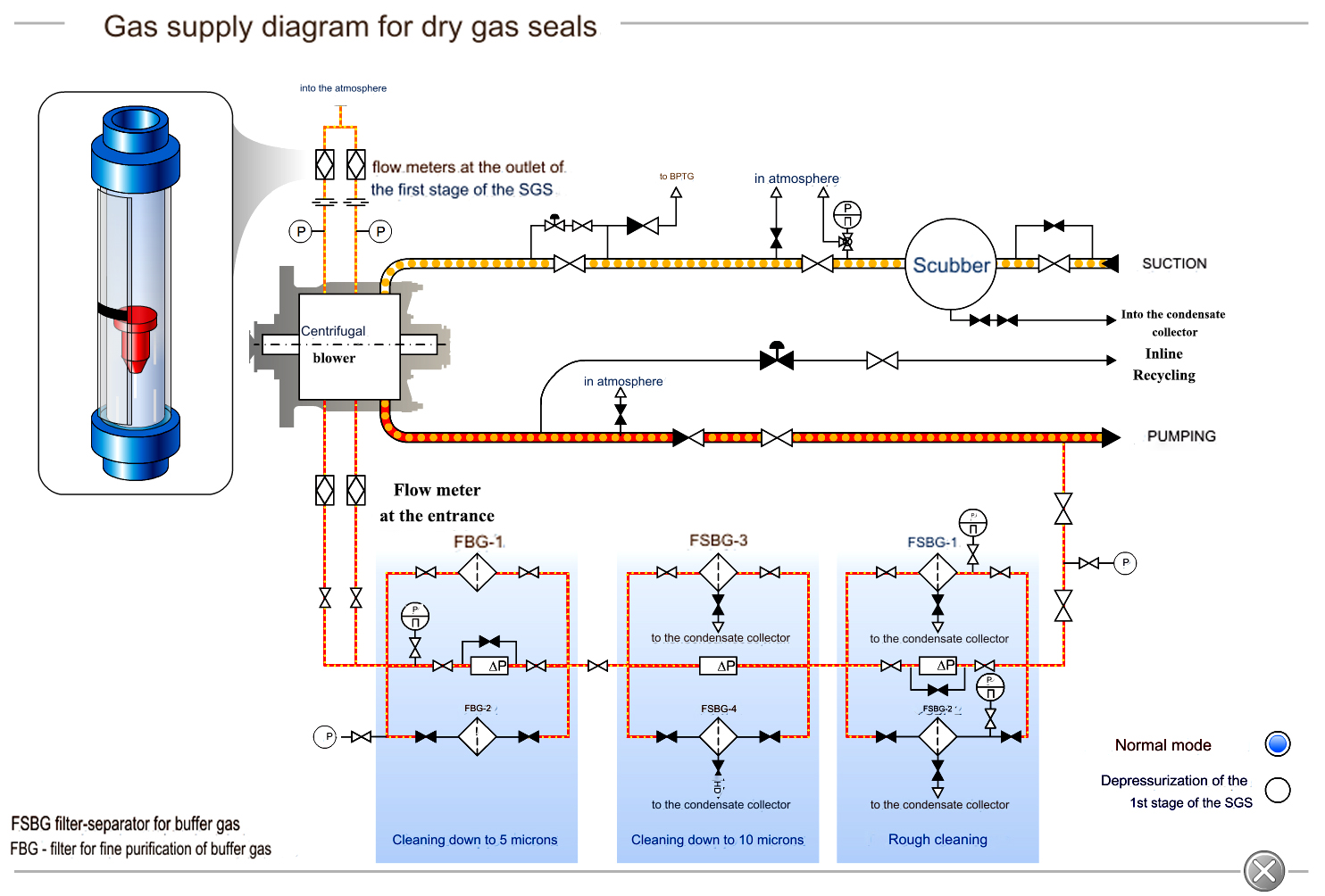

Supplement: Supplementary file 1 — Supplementary Information. [file 41598_2024_61361_MOESM1_ESM.zip › Translation of 1st Supplementary file in English.jpg]

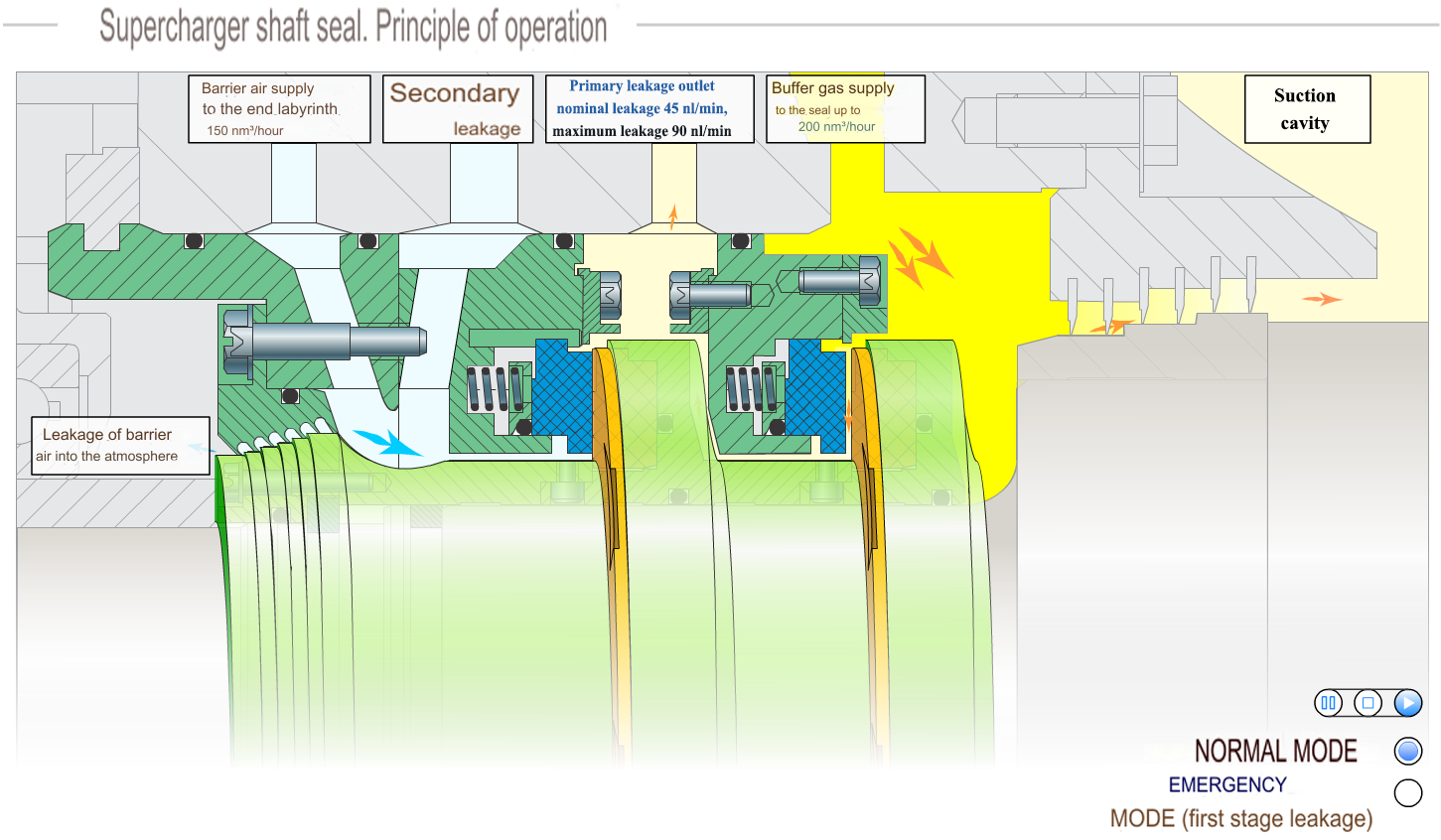

Supplement: Supplementary file 1 — Supplementary Information. [file 41598_2024_61361_MOESM1_ESM.zip › Translation of 2nd Supplementary file in English.png]
